# Post-biographical dignity in the age of artificial intelligence: Narrative, ePROMs and ethical challenges in end-of-life care

**DOI:** 10.1017/S1478951525100990

**Published:** 2025-10-27

**Authors:** Abel García Abejas, David Geraldes Santos, Helder Mota-Filipe, Àngels Salvador Vergés

**Affiliations:** 1 Núcleo de Estudos em Bioética (NEBUBI), Faculdade de Ciências da Saúde da Universidade da Beira Interior, Covilhã, Portugal; 2 Unidade de Medicina Geral e Familiar, Hospital Lusíadas Lisboa, Lisboa, Portugal; 3 Communication, Philosophy and Politics of the Faculty of Arts and Letters, University of Beira Interior, Covilhã, Portugal; 4 Department of Pharmacy, Pharmacology and Health Technologies, Faculty of Pharmacy, University of Lisboa, Lisbon, Portugal; 5 Board of Directors, Innohealth Academy, Barcelona, Spain

**Keywords:** Dignity, artificial intelligence, palliative care, digital legacy, post-biographical identity, ethics of technology

## Abstract

**Objectives:**

The growing integration of artificial intelligence (AI) and patient-reported digital tools (ePROMs and ePREMs) in palliative care offers new opportunities for personalised care yet also raises profound ethical and philosophical concerns. This paper examines how emerging technologies intersect with the concept of human dignity at the end of life, proposing an expanded notion of post-biographical dignity.

**Methods:**

Ethical-philosophical analysis based on critical readings of AI ethics, narrative medicine, and the philosophy of technology.

**Results:**

While digital tools such as ePROMs and ePREMs offer potential for richer, more person-centred care, they also risk reducing patients to data points and predictive profiles. Digital processes increasingly shape the narrative, vulnerability, and memory of the dying person. Post-biographical dignity calls for a reconceptualization of care that includes memory, relational continuity, and ethical engagement with digital remains.

**Significance of Results:**

End-of-life care in the age of AI must move beyond autonomy-focused ethics to encompass the narrative, relational, and posthumous dimensions of dignity. A critical, philosophically informed ethics is essential to prevent depersonalisation in digitally mediated care.

## Key messages

- Post-biographical dignity reframes personhood at the end of life by including digital identity and narrative persistence.
- ePROMs and ePREMs require an ethics grounded in relational dignity and continuity of narrative.
- Algorithmic care must be critically assessed to safeguard human uniqueness in palliative contexts.
- An interdisciplinary ethical framework combining phenomenology, narrative ethics, and digital health is needed.

## Introduction

The digitalisation of medicine is transforming not only clinical procedures but also the ontological categories through which we understand care, subjectivity, and death. This shift is critical in palliative care, where technologies such as artificial intelligence (AI) and patient-reported outcome systems (ePROMs and ePREMs) are reshaping how suffering is captured, interpreted, and addressed.

These tools promise greater personalisation, anticipatory care, and enhanced patient voice through structured data. However, they also risk depersonalising care, creating algorithmic opacity, and reducing narrative lives to quantifiable scores. As Kluge notes, digital health relies on mathematical, decontextualised representations that weaken patients lived experience (Kluge 2024).

This article introduces the concept of “post-biographical dignity” as a way of rethinking dignity beyond autonomy or physical integrity. Drawing on Byung-Chul Han, Giorgio Agamben, Paul Ricoeur and Harvey Chochinov, it argues for a model centred on narrative meaning, relational presence and the ethical management of the digital legacy (Agamben and Heller-Roazen 1998; Chochinov 2012; Han and Butler 2017; Ricoeur 1992). Dignity extends beyond the dying body to how life is remembered and preserved even posthumously, as noted by Shadbolt and Hampson (Shadbolt and Hampson 2024).

Predictive systems and “bare life” raise the ethical challenge of avoiding the patient’s reduction to a risk profile (Agamben and Heller-Roazen 1998).

Listening becomes an ethical act, not data collection, as Ricoeur suggests, identity is configured through narrative, especially in moments of profound vulnerability, a condition particularly salient in end-of-life contexts, where the self seeks recognition not only as a patient, but as a person whose story still matters, even in the face of digital mediation or after death (Ricoeur 1991, 1992). Post-biographical dignity reframes care as a form of memory protection in digital spaces where the person endures or is misrepresented.

## Digital subjectivity and the logic of data

In AI-assisted healthcare, the patient is no longer seen merely as a biological body, but as a datafile self: an entity represented, analysed, and anticipated through information flows. This ontological shift deeply reconfigures subjectivity, now mediated by algorithms, metrics, and predictive models. As Kluge notes, digital healthcare often operates through mathematical and decontextualised representations of patients, thereby weakening the comprehensive understanding of their experiences (Kluge 2024).

Tools like ePROMs and ePREMs are, in principle, designed to amplify the patient’s voice. However, when these data are filtered through AI systems, their function can shift. What began as a situated narrative of suffering can become a variable for optimization. Statements such as “I feel useless since I’ve been bedridden” may be transformed into a “depression risk” tag, stripping the comment of its biographical background. This reduction aligns with what Han calls the “violence of transparency”: the conversion of inner life into legible, quantifiable data, which erases ambiguity, silence, and existential complexity (Han and Butler 2017).

This algorithmic logic also operates anticipatorily. Predictive health systems intervene before symptoms arise, generating what Amoore calls an “ethics of the future,” a regime where present actions are justified by not-yet-manifested threats (Amoore 2013). In palliative care, this may lead to unsolicited interventions or therapeutic adjustments that are guided more by models than by the patient’s narrative.

Agamben foresaw this under the notion of “bare life”: a measurable, administered existence, excluded from ethical dialogue (Agamben and Heller-Roazen 1998). The person becomes an object of management, no longer an interlocutor. This not only alters clinical practice but also redefines the patient’s status in the system, from a biographical subject to a quantified profile.

This epistemic shift has critical implications. As Mittelstadt points out, algorithmic decision-making frameworks impose inferential logics that may not align with the values, temporalities, or meanings of those who are ill (Mittelstadt 2019). He calls this an “algorithmic epistemology”: a mode of knowledge that privileges correlation over comprehension.

Furthermore, as Deborah Lupton has shown, the rise of the quantified self-fosters internalised surveillance: the patient is not only observed but also learns to see themselves as a set of metrics and trends (Lupton 2014). This can lead to subtle forms of self-monitoring or algorithmic guilt (“if my data says I’ll worsen, what am I doing wrong?”), shifting responsibility from context to the individual.

Against these risks, it is essential to recover a notion of subjectivity that resists instrumentalization. Narrative medicine, as proposed by Charon, reconstructs the patient’s story as an ethical and clinical encounter (Charon 2006). In this framework, data does not replace the voice, but, if used sensitively, can accompany it.

This ontological shift echoes Heidegger’s distinction between *being* and *standing reserve* (*Bestand*) where the human is no longer encountered in their lived uniqueness but as a resource for data extraction (Heidegger 1977). In this light, patients risk becoming informational artefacts, available for manipulation rather than interlocutors in ethical dialogue. Post-biographical dignity then becomes a call to resist the reduction of the human to the calculable.

## Ethical ambiguities of ePROMs and ePREMs

Digital tools, such as ePROMs and ePREMs, are ethically ambiguous. On the one hand, they promote patient participation and claim to empower individual voices. On the other hand, they risk standardising, depersonalising, and flattening the very subjectivity they aim to capture. As Verhenneman notes, these instruments raise substantial concerns regarding data governance, context loss, and consent fatigue (Verhenneman 2025).

A deeper issue, however, is ontological: they often presuppose that lived experience is extractable, that it can be isolated from the biographical and embodied context of the person and analysed independently. However, as Greenhalgh reminds us, the self is always embedded in a narrative, a story that includes memory, intention, and fragility (Greenhalgh 2009). To dislocate suffering from its story is not just a technical error; it is a form of misunderstanding.

This reduction of lived experience to extractable data aligns with what Michel Foucault described as *biopower* – the modern regime in which institutions manage and optimize life not through repression, but through subtle mechanisms of surveillance, normalization, and productivity. In the context of digital healthcare, biopower takes a new form: algorithmic authority. Patients become subjects of constant optimization, monitored and adjusted according to predefined metrics and clinical expectations. Tools like ePROMs, while promising participatory care, may paradoxically reinforce this dynamic by converting the patient’s narrative into a site of evaluative control. The risk is that care becomes conflated with compliance and listening turns into a form of soft surveillance. Palliative care, rooted in relational ethics, must resist this logic of governability and affirm patients’ right to ambiguity, interpretive depth, and the irreducibility of their suffering (Foucault 2003).

The structural pressures of clinical systems, time constraints, digital overload, and performance metrics, make it tempting to use ePROMs as interpretive shortcuts. This can lead to what we might call ethical reductionism through measurement, where complex forms of distress are translated into standardised categories without being truly heard.

Efforts such as “Design for Values in AI Systems” aim to embed ethical principles during the development phase of technologies (Buijsman 2024). Likewise, broader frameworks of value-sensitive design have highlighted the need to move beyond functional optimisation toward moral reflection (van den Hoven et al. 2015). However, these approaches remain insufficient if they overlook the existential dimensions of illness, dying, and care. Digital tools must not only be respectful in a procedural sense, but they must also be attuned: capable of acknowledging opacity, silence, and ambiguity.

Moreover, as Sharon has shown, the increasing responsibilities of patients through digital self-monitoring can lead to guilt, self-surveillance, and internalised performance pressure, dynamics particularly problematic in palliative contexts (Sharon 2017).

An ethics of care in the age of digital medicine must resist the impulse to convert human complexity into data legibility. ePROMs may be helpful instruments, but only if embedded in relationships that listen before measuring and understand before categorising.

## Dignity, narrative, and algorithmic listening

Dignity, particularly at the end of life, cannot be reduced to legal status or abstract principles. It is above all a form of being recognised and heard in one’s vulnerability. Harvey Chochinov has defended this idea through his Dignity Therapy model, where the patient’s narrative becomes a space of meaning and identity restoration (Chochinov 2012).

This limitation is especially critical in palliative care, where often what matters is not what is said, but how, when, and what cannot be said. The language of suffering is made of pauses, repetitions, glances, and absences. For Ricoeur, this form of presence with the other constitutes a “solicitude,” an ethical act of receiving another’s fragility (Ricoeur 1991, 1992). Cavarero further argues that our identity is not only self-narrated but shaped by how others recount and receive our story, especially in conditions of vulnerability and dependence (Cavarero 2000).

However, when listening is mediated by algorithmic systems, this relational dimension is transformed. Algorithms do not listen in a hermeneutic sense; they process, classify, and predict. As Boddington notes, although AI systems can simulate understanding, they lack moral depth: they do not interpret meanings, perceive metaphors, or engage with silence (Boddington 2023).

Emmanuel Levinas deepens this ethical horizon by asserting that the encounter with the Other begins not with knowledge or classification, but with a face that calls us into responsibility. This demand precedes cognition and resists objectification. In the presence of the dying person, this face is not only physical, but narrative, fragile, and vulnerable. It calls for an attentiveness that no algorithm can replicate. When digital tools mediate clinical listening, there is a danger that this primordial ethical appeal is silenced or bypassed. Defending dignity, then, means preserving the asymmetry of the ethical encounter, where the clinician is first a listener, not an interpreter, and where care is rooted in responding to suffering rather than analysing it (Levinas 1969).

Listening must be radically rethought. It is not enough to capture voice data or free text. Listening requires openness, a willingness to be affected, and an ethical stance that does not equate every opacity with information. Recent work in tech ethics advocates a shift from data-centric AI to relationship-centred AI, especially in contexts of suffering and moral complexity (Durán et al. 2022).

Furthermore, the push to make listening more efficient, automating anamnesis, and optimising communication risks emptying clinical care of its ethical depth. As Svenaeus observes, medicine becomes dehumanised when it forgets that its centre is not the disease, but the human suffering interpreted from within (Svenaeus 2000).

If we are to preserve dignity as something recognised, digital tools must be integrated without replacing the narrative and relational dimension of care. In the context of AI, listening must not mean merely “processing input text.” It must mean ethically engaging with the singularity of the speaker, even when their expression escapes the language of algorithms.

## Digital identity and post-biographical dignity

Death no longer marks the end of identity. In the digital age, a person’s traces persist across various platforms, including active profiles, shared memories, metadata, recordings, and even chatbot interactions. This persistence raises radical ethical questions in end-of-life care: who manages the narrative of the deceased? Can their memory be manipulated? Do algorithms have the power to represent, or distort, a life beyond its biological end?

Shadbolt and Hampson have explored this phenomenon through the idea of “digital remains,” showing how emerging technologies not only prolong symbolic presence but also reshape mourning, memory, and even relational simulations using AI (Shadbolt and Hampson 2024). These practices shift how we relate to death, blurring the boundary between presence and absence.

Jacques Derrida’s concept of *hauntology* offers a philosophical lens to understand this blurred boundary. In his view, the dead are not entirely gone; they haunt the present as spectral presences, neither absent nor fully present, that demand ethical attention. Digital remains intensify this haunting: profiles, messages, and AI reconstructions continue to speak in the name of someone who can no longer consent, clarify, or contest. Post-biographical dignity, then, is not merely about remembering the deceased but about relating responsibly to these spectral traces. It requires ethical discernment about which presences we perpetuate, who narrates them, and for what purposes (Derrida 1994).

In this context, we propose the notion of post-biographical dignity: an expanded form of dignity that encompasses not only the body and consciousness while alive, but also the representation, remembrance, and ethical handling of digital identity after death. This builds upon a critical reading of rights to memory, to be forgotten, and to consented representation, areas still underdeveloped in contemporary bioethics (Lei et al. 2025).

The right to post-biographical dignity includes, for example, the ability to decide whether one’s digital data may be archived, transformed, or deleted. It also includes the right not to be “recreated” by AI without explicit consent, a growing phenomenon in commercial and family-based applications (Lagerkvist 2017).

Moreover, dignity in this sense is not only individual. As Floridi notes, digital identity is relational, co-constructed in dialogue with others and sustained by socio-technical infrastructures. Thus, the ethics of digital legacy must consider not only the individual’s will but also the communities, emotional, professional, and cultural, to which the person belonged (Floridi 2013).

In palliative care, these questions are not peripheral. They can affect how patients feel heard, remembered, or even betrayed by the technologies surrounding their final days. If technology aspires to be truly compassionate, it must respect not only the life that ends, but the one that continues to be represented through symbols, images, texts, and systems.

Post-biographical dignity does not seek to eternalize the person, but to protect their story beyond biological silence. It requires asking not only how we die, but also how we continue to be interpreted when we can no longer respond.

## Philosophical critique of technological reductionism

The growing integration of artificial intelligence into medicine has reinforced a technical vision of care that identifies clinical excellence with the capacity to measure, predict, and manage. However, this framework carries the risk of technological reductionism, where the complexity of human suffering is reduced to an optimisation algorithm.

Vincent C. Müller reminds us that the rise of AI redefines foundational categories such as decision, action, and relation (Müller 2016). In palliative care, this may manifest as a technologization of dying, where information flow management replaces symbolic, ethical, and affective accompaniment.

Byung-Chul Han describes this dynamic as characteristic of the performance society, where even death must be efficient, scheduled, and monitored (Han and Butler 2017). The pressure to fit the model can lead to death being experienced not as a human transition, but as a clinical output, generating a subtle form of violence against those who do not conform.

Here, hermeneutic philosophy becomes a critical tool. As Borgmann argues, modern technology not only transforms actions but shapes our frameworks of meaning, what we consider visible, valuable, or worthy of attention (Borgmann 1984). The data paradox is that the more we measure, the more we risk ignoring what does not fit system formats.

Tolerating uncertainty, embracing ambiguity, and being open to silence are deeply clinical gestures. As Gadamer reminds us, medicine is not an exact science, but a practical art grounded in judgment, presence, and understanding of the other in their singularity (Gadamer 1996). This contradicts the notion that more data automatically leads to better care.

Moreover, technological reductionism can have harmful effects, including overtreatment, the automation of therapeutic relationships, or even moral deskilling (“the algorithm said so”). These risks demand a critical ethics, not centred on abstract principles but on situated reflection about what it means to care in liminal situations (Wang and Hsu 2023).

Thus, defending a philosophy of care is not about opposing technology, but rather resisting its absolutisation. As Ricoeur puts it, the self is not something to be calculated but a story that must be heard and accompanied (Ricoeur 1991, 1992).

## Key points for an ethics of care in the age of artificial intelligence

Before concluding, it is helpful to summarise several core ideas that should guide the development and application of digital technologies in palliative care. These are not formulas, but rather ethical and philosophical reminders to maintain a focus on the human dimension of care.

- Dignity encompasses not only autonomy and physical comfort, but also memory, narrative identity, and symbolic continuity beyond death.
- Clinical listening cannot be automated: interpreting suffering requires presence, contextual attention, and openness to silence.
- ePROMs and ePREMs are valuable tools, but incomplete: they must complement the therapeutic relationship, not replace it.
- Digital identity is an integral part of a person; ethical handling requires consent, the right to be forgotten, and fair posthumous representation.
- Algorithmic reductionism can obscure what truly matters: not everything that matters in palliative care is measurable or predictable.
- Narrative medicine remains central because to dignify is also to listen and sustain the story of the other, even in its fragmentation.
- An ethics of care requires a relational revision: it must embrace fragility, reciprocity, and resistance to the technification of human connection.

## Conclusion

The integration of artificial intelligence into palliative care is profoundly transforming not only clinical practice but also our understanding of dignity, listening, and identity. In response to technological optimism, this paper proposes a critical, ethical, and philosophical approach: the need to consider post-biographical dignity as a relational, narrative, and digital extension of the human.

End-of-life care cannot be reduced to symptom relief or data management. It involves welcoming stories, attending to silences, and recognizing the symbolic persistence of the person even after death. As Ricoeur reminds us, ethical recognition requires seeing the other not merely as a sick body, but as a subject of speech and memory (Ricoeur 1991, 1992).

This emerging horizon calls for a revision of traditional bioethical frameworks, which have focused heavily on decisional autonomy and informed consent. Instead, we must move toward a relational and hermeneutic ethics of care, incorporating interpretation, fragility, digital representation, and symbolic legacy (Tronto 1993).

Moreover, digital tools, ePROMs, chatbots, predictive algorithms, must be evaluated not only in terms of effectiveness but also of ethical sensitivity. As Vallor argues, truly responsible AI in vulnerable contexts requires transparency, meaningful human oversight, and openness to critical scrutiny (Vallor and Vierkant 2024).

Honouring dignity in the age of AI does not mean rejecting technology, but ensuring it serves a more humane, complex, and situated form of care. No dataset can replace a life story, and no predictive model can determine how a person should be remembered.

As philosopher Adriana Cavarero reminds us, the human being is above all a narrated being, someone whose uniqueness emerges when another voice chooses to tell their story with care. In this context, identity is not only self-narrated but shaped by how others recount and receive our story, especially in conditions of vulnerability and dependence (Cavarero 2000). The challenge of AI in palliative care is not to narrate better, but to never stop listening.

This ethical imperative invites us to move beyond technical assessments of digital tools and ask more profound questions: What kind of future are we building through these systems? Who gets to decide which lives are preserved, narrated, or optimized? Moreover, how do we ensure that dignity, especially in its most vulnerable, voiceless, or posthumous forms, remains protected in the face of accelerating automation? In this sense, post-biographical dignity is not only a clinical or technological issue, but a metaphysical and political stance. It demands that we reimagine care not merely as intervention, but as responsibility for memory, representation, and the symbolic continuity of persons who can no longer speak. To care, ultimately, is to become the custodian of another’s story, not to complete it, but to honor its unfinished humanity.
